# A Hybrid Stochastic Approach for Self-Location of Wireless Sensors in Indoor Environments

**DOI:** 10.3390/s90503695

**Published:** 2009-05-15

**Authors:** Jaime Lloret, Jesus Tomas, Miguel Garcia, Alejandro Canovas

**Affiliations:** Departamento de Comunicaciones, Universidad Politécnica de Valencia. Camino Vera s/n, 46022, Valencia, Spain; E-Mails: jtomas@dcom.upv.es; migarpi@posgrado.upv.es; alcasol@epsg.upv.es

**Keywords:** indoor location system, positioning system, WLANs

## Abstract

Indoor location systems, especially those using wireless sensor networks, are used in many application areas. While the need for these systems is widely proven, there is a clear lack of accuracy. Many of the implemented applications have high errors in their location estimation because of the issues arising in the indoor environment. Two different approaches had been proposed using WLAN location systems: on the one hand, the so-called deductive methods take into account the physical properties of signal propagation. These systems require a propagation model, an environment map, and the position of the radio-stations. On the other hand, the so-called inductive methods require a previous training phase where the system learns the received signal strength (RSS) in each location. This phase can be very time consuming. This paper proposes a new stochastic approach which is based on a combination of deductive and inductive methods whereby wireless sensors could determine their positions using WLAN technology inside a floor of a building. Our goal is to reduce the training phase in an indoor environment, but, without an loss of precision. Finally, we compare the measurements taken using our proposed method in a real environment with the measurements taken by other developed systems. Comparisons between the proposed system and other hybrid methods are also provided.

## 1. Introduction

Currently, sensor networks are the main part of many monitoring and control systems. Many of them tend to be wireless because it allows them to be spatially distributed. Wireless Sensor Networks (WSNs) [1] are formed dynamically because the connectivity between nodes depends on their position and their position variation over the time. These kinds of networks are easy to be deployed and are self-configuring. A sensor node is a transmitter, a receiver, and it offers services of routing between nodes without direct vision, as well as recording data from other sensors.

Since the 1950s, location systems have been incorporated into our lives. In the 1980s the Global Position System (GPS) [2] began as a location method for outdoor environments. This system is based on a triangulation system of variables, where we know the position of a device thanks to the existing satellite network. Now, this system remains the most used because of its good performance and the low price of the required devices. At the end of the year 2000 other location systems appeared, based on cellular networks [3]. These systems are designed for emergency situations. In this case, the base stations are used as a reference point and the location is made through the distance and the angle of the signal. These systems are developed to work in outdoor environments and the devices must also have several communication skills.

Previous technologies are not adequate for indoor environments. This is mainly due to the signal characteristics. Other wireless technologies, such as IEEE 802.11a/b/g [4], Radio Frequency IDentification (RFID) [5], Ultra Wide Band wideband (UWB) [6], Bluetooth [7] or Zigbee [8], must be used for indoor locations. The main problem in these environments is the multipath effect and signal variability. The location in these types of environments can be centralized [4] or distributed [9]. The centralized location uses reference devices. These devices tend to have a greater capacity and they are located in a fixed position, for example a base station (BS) or an Access Point (AP). In contrast, in the distributed location there are no reference devices. The devices interact with their neighbors in order to know their position.

A study about the oscillation of the received signal is shown in reference [10]. The RSS variation is introduced in our approach in order to obtain smaller degree errors in the location service. The three main issues that make variations in the RSS are the following ones:

- Temporal variations: when the receiver remains in a fixed position, the signal level measured varies as time goes by.
- Small-Scale variations: the signal level changes when the device is moving over small distances (less than the wavelength). In IEEE 802.11 b/g technologies the wavelength is 12.5 cm.
- Large-Scale variations: the signal level varies with the distance due to the attenuation that the radio frequency (RF) signal suffers with the distance.

Besides these typical variations of the RF signal together with the receiver mobility, we have also considered the temperature and humidity variations, the effect of opening and closing doors, the changes in the localization of the furniture, and the presence and movement of human beings, which are all characteristics of indoor environments. These variations have already been analyzed in [11].

All these systems can be used in different applications. Localization in sensor networks has attracted a large research effort in the last decade. Some WSNs location systems application areas are:

- Emergencies: When we want to locate an individual in the case of an emergency (injury or criminal attacks) or in a life-threatening situation. Both can be located using the positioning capability of the mobile device.
- Information: It can be used in public places such as swimming pools, museums, conferences, etc. in order to provide information service about this place to the user depending on his position.
- Navigation: When the user needs to meet the situation of addresses, positions, directions in an indoor places such as big supermarkets, commercial centers, etc.
- Discovery: When it is necessary to find or locate things or persons in indoor places. It is very useful to locate people with Alzheimer or to locate disabled people with very little motion.
- Security: It can be used to avoid theft, to move unwanted items, etc. Wireless sensors would be in specific places, when the sensors transfer a position threshold they send an alarm.
- Tracking: When it is required to track a device or person inside a building.

There are two main methods to estimate the position in indoor environments. On the one hand, there are the so-called deductive methods. These take into account the physical properties of signal propagation. They require a propagation model, topological information about the environment, and the exact position of the base stations. On the other hand, there are the so-called inductive methods. These require a previous training phase, where the system learns the signal strength in each location. The main shortcoming of this approach is that the training phase can be very expensive. The complex indoor environment makes the propagation model task very hard. It is difficult to improve deductive methods when there are many walls and obstacles because deductive methods work estimating the position mathematically with the real measures taken directly from environment in the training phase [12].

In this work we present a hybrid location system using a new stochastic approach which is based on a combination of deductive and inductive methods. This system has been developed for wireless sensor networks using the IEEE 802.11b/g standard in order to use a deployed wireless access network that is also used for internet access and data transfer. On the other hand, the aforementioned technology allows us to cover a hard indoor environment without many base stations. The goal of this work is to reduce the training phase without losing precision.

The remainder of this paper is organized as follows. Section 2 presents the best known related work on location methods in sensor networks. Our hybrid location system is described in Section 3. Section 4 shows the efficiency of our system. It shows real measurements and compares our proposal with other systems. A comparison between our proposal and other hybrid systems proposed in the literature is shown in Section 5. Section 6 concludes the paper and discloses our planned future work.

## 2. Related Works

The main location systems in WSNs are based either on the GPS [2], on localization algorithms based on different measurement techniques [13] or systems based on known sensor positions [14]. The system proposed in reference [14] uses the physical layer and the Medium Access Control (MAC) to transmit location information between pairs of sensors using IEEE 802.15.4.

The well-known analytical techniques used in localization algorithms are the angle of arrival (AOA), the time of arrival (TOA) and the time difference of arrival (TDOA) [15-17], relative distance [18] and the RSS [13]. This section discusses some works related with deductive and inductive location systems and other hybrid location systems.

### 2.1. Deductive Methods

As shown in reference [4], the effectiveness of the measurement techniques based on location algorithms in indoor environments is limited by multiple reflections. That paper describes the RADAR system which is based on RF. The system uses the received signal strength information for a trilateration system and signal propagation models to locate the device. While the empirical model has higher precision, the signal propagation method is easier to use.

In [14], measurement based statistical models of TOA, AOA and RSS are presented and used to generate localization performance boundries. Such boundries are useful as design tools to help choose among measurement methods, select neighborhood size, set minimum reference node densities, and compare localization algorithms.

R. Schmidt proposed, in reference [15], a system that processes the signals received from the emitter on an array of sensors. The system considers sensors with arbitrary locations and arbitrary directional characteristics (gain/phase/polarization) in a noise interference environment of arbitrary covariance matrix. The author proposes the multiple signal classification (MUSIC) algorithm to determine the parameters of multiple wave fronts arriving and then the method calculates the position.

A paper where the authors explain another trilateration based location system is [19]. Niculescu and Nath present an indoor positioning architecture that does not require a signal strength map, simply requiring the placement of special VOR (VHF Omnidirectional Ranging) Base Stations (VORBA). The accuracy of the positions obtained by their system, of 2.1 meter median error, is comparable to the original RADAR. Its basic idea is to find the strongest maximums in the signal strength, and use them as the most likely directions in which the device can be placed.

Reference [20] describes two approaches developed by the same authors of this paper, where wireless sensors could find their position using WLAN technology. The scenario is an indoor environment that contains walls, several sources of interferences, multipath effects, humidity and temperature variations, etc. Both approaches are based on the RSS. The first approach uses a training session and the position is calculated based on a heuristic model using the data obtained from the training session. The second approach uses a triangulation model with some fixed access points and a signal propagation model based on wall looses to calculate the position.

The techniques based on RSS are easier to implement. This is due to the fact that standard wireless devices possess features for measuring this value. As indicated in [21], we must take into account that the location error in triangulation systems is very low when the number of base stations is higher than seven and the triangulation analysis is in three dimensions. As we have said previously, when the number of base stations is high, the location system provides more precision, but it makes the sensor estimate more distances (for every base station), so the sensor could need more processing time, diminishing the overall system performance [20].

### 2.2. Inductive Methods

Inductive methods use location techniques based on RSS profiles. This technique consists of building a map according to the signal strength behavior with respect to the coverage area. A sensor location can be estimated with the gathered information from several base stations. These access points or base stations work with the signal strength vector. The vector is obtained from the RSS model and probabilistic techniques or various methods based on neighbors. With this information the system can estimate the possible area where the sensor could be located.

This method uses two parameters: a) the likelihood that an object is in this area and b) the precision of the signal strength. This second parameter depends on, for example, the size and the type of the location area. With these types of systems, the final user does not require any additional hardware for the localization process. These algorithms give very low localization errors using the IEEE 802.11 technology [21]. According to reference [21], we can consider three different techniques used in algorithms based on patterns or areas: a) single point adaptation, b) likelihood based on areas and c) Bayesian networks.

There are statistical models based on the signal strength, where the distance between different sensors is obtained by the calculation of a Cramér-Rao bound (CRB) on the location estimation precision possible for a given set of measurements (see reference [14] for more details). This is a useful tool to help system designers and researchers select measurement technologies and evaluate localization algorithms.

Reference [22] shows a localization system based on the RSS, which considerably reduces the number of broadcasting stations. This system is called Location Estimation Assisted by Stationary Emitters (LEASE) for indoor RF wireless networks and uses a few stationary emitters and sniffers in a novel way to solve the location estimation problem. The estimation engine uses non-parametric modeling techniques that automatically capture the anisotropy of the RSS encountered in indoor environments.

Siddiqi *et al.* present in [23] another work where we can see the use of Bayesian networks. In this paper, the authors use a robot to retain samples. These samples are used to know the location by means of a probability density function. Each time the robot moves or senses the signal strength of an AP, a Bayes filter is used to recursively update the belief function (Monte Carlo localization (MCL) algorithm). Their results show that accurate localization (∼ 2 m) is achieved in most test cases and the average localization error decreases with time.

Another important inductive location system is LANDMARC (see reference [5]). It is a localization prototype that uses RFID technology to locate objects inside the buildings. Although RFID is not designed for indoor location, the authors demonstrate that active RFID is a viable and cost-effective candidate for indoor location. References [4,13,20,21] can be consulted to examine some inductive methods in depth.

### 2.3. Hybrid Methods

There are other papers where the authors propose hybrid systems. In [24], the authors propose a radiolocation scheme based on the AOA and the TOA in multipath environments with a single base station. This scheme is used in macrocellular networks (such as the Code Division Multiple Access cellular network) and Global System for Mobile communications (GSM) networks. In [25], the authors combine RSS measurements and the TDOA measurements. This model is sturdy to variations of measurement noise and quantization. The error is lower than ones based on individual measures.

There are few works related with the hybrid location techniques in sensors networks. In reference [26] Cramer-Rao Bound (CRB) is used for location estimation using of two different hybrid schemes: TOA/RSS and TDOA/RSS. These techniques provide improved location accuracy with respect to TOA and TDOA schemes for networks with devices having communications ranges of 30 meters or less.

In [27] Sahinoglu and Catovic developed a hybrid location estimation scheme for heterogeneous WSNs with unsynchronized short range simple relays and mobile sensor nodes, and synchronized stations. In this work, the authors use RSS measurements as well as TOA and TDOA measurements. These measurements are used to filter out the clock offset that appears due to the lack of synchronization. They quantify the estimation accuracy of the scheme by deriving the Cramer-Rao bound (CRB), and discuss the performance trade-off between the number of synchronized and non-synchronized devices involved. This work takes into account the heterogeneity of sensor networks, in terms of communication range, time synchronization and routing capabilities of network devices.

If we analyze the hybrid location systems in WLAN, regardless of whether they are sensor networks or any network, there are several proposed systems. In [28] the authors propose a hybrid location system based on three stages. Firstly, it establishes a database that contains a distance-signal strength map. Next, the system uses the database to obtain the distances between mobile terminal and base stations. Thirdly, this proposal applies trilateration to calculate the mobile terminal position. In this case, we see that the hybrid modeling has better accuracy than propagation modeling.

Finally, another hybrid location method is proposed in [29]. This hybrid method has two stages. In the first stage, it uses the fingerprinting method with a fast training phase to obtain an estimate of the mobile user (MU) position. In the second stage, trilateration is used to compute the MU location more accurately. The result shows their proposed method is better than the simple trilateration method based on general propagation mode, but worse than the fingerprinting method with a medium training phase.

The Euclidean models are optimum when there are multiple access points. Although some works show that the statistical properties of the RSS signal is stationary under certain circumstances, the distribution of the RSS is not usually Gaussian, it is often left-skewed and the standard deviation varies according to the signal level. Signals from multiple APs are mostly independent and the interference from other APs using the same frequency does not have a significant impact on the RSS pattern. Consequently, the coverage areas can be grouped together as a group of clusters. More than one cluster may represent one location because of the multimodal distribution of the RSS. In such a case, using a simple Euclidean distance to determine the location may easily classify some patterns into a wrong location. Our proposal combines the advantages of the deductive and inductive methods in order to provide more accurate measurements in hard environments (few base stations and/or few trained points).

## 3. Hybrid Stochastic Approach to Location Estimation

In this section we explain the mathematical assumptions used in our proposal. We analyse the inductive and deductive methods from a statistical point of view. In this way, we can describe our hybrid model. Table 1 shows the variables used in the analysis.

### 3.1. Stochastic Approach for Location Estimation

The location estimation problem can be statistically stated as follows. For simplicity, the true distribution Pr(X = x) and Pr(X = x | Y = y) are denoted as Pr(x) and Pr(y). The model parameters are denoted by p( ).

Let *b* be the number of base-stations. We denote *o* as an observation. The observation variable is a *b*-dimensional vector; one for each signal strength from each base-station. We denote as *o^j^* the signal strength from base-station *j* for *j* ∈ {1,…,*b*}. We have a location *l* associated to each observation. In this work we use bi-dimensional locations for simplicity, but it can be used in three dimensions easily.

The methodology used is based on the definition of a function Pr(*l*|*o*) that returns the probability of the location *l*, given the observation *o*. This nomenclature has been used in other proposals [22, 28, 29]. Once this function is estimated, the problem can be formulated to find the location *l* that maximizes the probability Pr(*l*|*o*) for a given observation *o*. Using Bayes' theorem, we can write:

(1) $$\mathrm{Pr}(l|o)=\frac{\mathrm{Pr}(l)\mathrm{Pr}(o|l)}{\mathrm{Pr}(o)}$$

The denominator in equation (1) does not depend on the location variable *l*. And, therefore, the location estimation problem can be presented as:

(2) $$l\prime =\underset{l}{\arg\max}\mathrm{Pr}(l)\mathrm{Pr}(o|l)$$

where Pr(*l*) is the *prior probability* of the location *l*, knowing the observation. This probability can be used to incorporate information such a more training locations [22] or tracking [29] to our statistical model. The tracking information will not be taken into account in this work, so, for the prior probability, we use the uniform distribution.

In equation (1), Pr(*o*|*l*) is the so called *likelihood function*. It estimates the probability of one observation given a location. In the literature, we find two main approaches to estimate this function: inductive approach and deductive approach. The next two subsections explain these methods analytically in order to propose our approach.

### 3.2. Inductive Approach for Location Estimation

On the one hand, the *inductive approach* estimates the *likelihood function* measuring directly the signal strength in each place. That is, several measurements are taken for each training place; then, the function p(*o*|*l*) is estimated. The main drawback of this approach is the time consuming nature of the training phase. We denote as *T* the set of training data, formed by *t* observations with their respective locations. Each training data, *T_i_*, it is represented as (*l_i_*, *o_i_*), where *i* can be from 1 to *t*. Several alternatives has been proposed in the literature to estimate p(*o*|*l*) from *T*: the histogram method [30, 32], the Bayesian method [33] or the kernel method [30, 34]. Another drawback is that this model only returns one of the locations from the training set. In order to solve this problem, several proposals can be found in reference [35].

### 3.3. Deductive Approach for Location Estimation

On the other hand, the *deductive approach* estimates the *likelihood function* by using empirical formulas about the signal propagation in an indoor environment. In this approach, we need to know the location of each base-station, the described map of the environment (walls, obstacles, etc.) and a propagation model. Several propagation models can be seen in references [35, 36]

If we assume that each observation *o^j^*, from the vector o, is mutually independent, we can write:

(3) $$\mathrm{Pr}(o|l)=\prod_{j=1}^{b}\mathrm{Pr}(o^{j}|l,B^{j})$$

where *b* is the number of base-stations; *B^j^* is the base-station *j*; and, *o^j^* is the observation signal strength from base-station *j*. In our study the base-station *B^j^* is characterized by two variables (*l^j^*, *o_0_^j^*). The variable *l^j^* denotes the location of base-station *j*, and o*_0_^j^* denotes the mean signal strength measured to *d_0_* distance from base-station *j*.

In this work, we assume that (*o^j^*|*l,B^j^*) follows a Gaussian distribution with standard derivation σ. The following empirical propagation model, which supposes that signal strength is measured in dB, is used [36, 37]:

(4) $$P(o^{j}|l,B^{j})\approx N(o_{0}^{j}+10n\log(\frac{d^{j}}{d_{0}})+L_{w}^{j},\sigma^{2})$$

where, *d^j^* is the Euclidian distance between the observation location (*l*) and the base-station *j* (*l^j^*), *n* is the attenuation variation index (*n* value depends on the specific propagation environment) and *L_w_^j^* is the attenuation caused by the obstacles. In our study the value of *L_w_^j^* depends on the number of walls that the line of sight crosses from the base-station *j* to the location. We will use *L_w_^j^* = *wL_0_*. Where *w* is the number of wall crossed and *L_0_* is the wall average attenuation. Finally, let *N*(*μ*, *σ*^2^) be the normal distribution with mean *μ* and variance *σ*^2^.

### 3.4. Stochastic Hybrid Approach for Location Estimation

In the inductive approach we assume that the signal distribution for each training sample location is known in advance. Taking a sample for all possible locations is not a realistic assumption. However, for a given location we can have several training samples near to our location. In the hybrid approach we are interested in combining the information of both previous approaches to improve the system. That is, we know the signal distribution for several training samples near our location and we know how the signal is attenuated from the location of these samples to our actual location. Without loss of generality, we can write:

(5) $$\mathrm{Pr}(o|l)=\sum_{i=1}^{t}\mathrm{Pr}(T_{i}|l)\mathrm{Pr}(o|l,T_{\text{i}})$$

We assume that Pr(*T_i_*|*l*) is uniformly distributed. Then, we are only interested in defining the second term. Using the same assumption that in equation (3), we can write:

(6) $$\mathrm{Pr}(o|l,T_{i})=\prod_{j=1}^{b}\mathrm{Pr}(o^{j}|l,B^{j},T_{i})$$

Now, we define the random variable (*o^j^*|*l,B^j^*,T_i_) in the same manner as (*o^j^*|*l,B^j^*) has been defined in equation (4). But, instead of *o_0_^j^* (the signal strength measured in the reference distance *d_0_*), we use *o_i_^j^* (the signal strength measured in the training sample location *i*).

(7) $$(o^{j}|l,B^{j},T_{i}^{j})=o_{i}^{j}+10n\log(\frac{d^{j}}{d_{i}^{j}})+\frac{{L_{w}}^{j}}{{L_{wi}}^{j}}$$

where *o_i_^j^* is a random variable that represent the signal strength of training sample *i* from base-station *j. d_i_^j^* is the distance from training sample *i* to the base station *j*. And, *L_wi_^j^* is the wall attenuation from training sample *i* to the base station *j*. Note that in this equation, *X_σ_* has been eliminated because the variability is included in the random variable *o_i_^j^*.

### 3.5. Implementation Details

From equation (7) the random variable *o_i_^j^* can be expressed as follows:

(8) $$o_{i}^{j}=o^{j}-10n\log(\frac{d^{j}}{d_{i}^{j}})+\frac{{L_{w}}^{j}}{{L_{wi}}^{j}}$$

**Figure 1.**
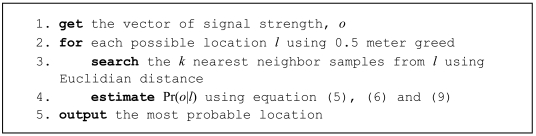
Proposed algorithm.

In the training phase, we have estimated p(*o_i_^j^* |*l_i_,B^j^*). In this stage several methods such as histogram or kernel can be used. Then, using equation (8), we can write:

(9) $$\text{p}(o^{j}|l,B^{j},T_{i}^{j})=\text{p}(o^{j}-10n\log(\frac{d^{j}}{d_{i}^{j}})+\frac{{L_{w}}^{j}}{{L_{wi}}^{j}}|l,B^{j})$$

where n = 2 in free space. In order to obtain the optimal location for equation (2), the proposed algorithm is written in pseudocode in Figure 1. Its explanation is as follows: given the input signal strength the location probability for each point is evaluate using 0.5 meter greed. For each point the k nearest samples are taken. The probability of this location is calculated using equation (5), but, using only these k samples instead the all training set. Farther samples will distort the results. For each sample (of k nearest samples), first we use equation (9) to combine the deductive approach, to take into account the shift from the actual location to the sample location, and, second the inductive approach, to obtain the signal probability in a well known place.

## 4. Experimental Results

This section shows the results obtained from a real environment to test our proposal. First, we will test the errors based on the number of samples and based on the number of base stations. Then, we compare it with other commercial and implemented location systems.

### 4.1. Test Bench

**Figure 2.**
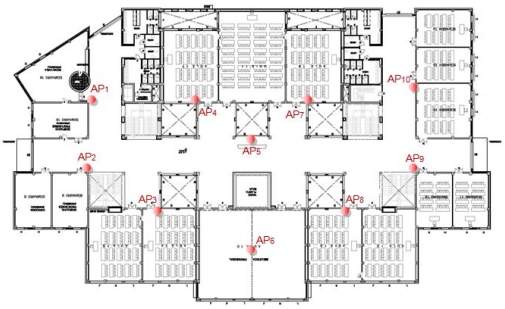
Test bench place used in the experiments.

To assess our proposal, we have deployed the approach in an indoor wireless environment. This place is located on the first floor of the “A building” in the “Campus Gandia” of the Polytechnic University of Valencia. The distribution of this floor is shown in Figure 2. There are 10 access points acting as base stations. These base stations have a fixed position and their transmission power is known in advance.

### 4.2. Error Measurement Based on the Number of Samples

Our proposal takes into account k nearest neighbour samples from a position using Euclidian distance (see Figure 1). This experiment gives us the optimum number of samples in order to obtain the lowest error. In order to test our approach we took 56 samples spread equitably throughout the floor of the building. The floor was split in a grid where sampling points are placed every 2 meters.

**Figure 3.**
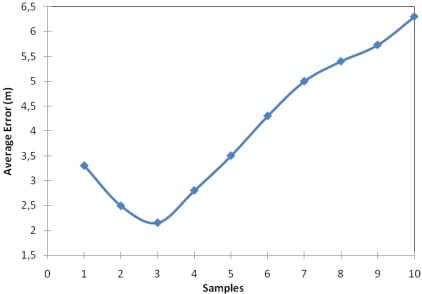
Average location estimation error as a function of the number of samples.

Figure 3 shows that the error is not reduced linearly as regards the number of samples. It has an inflection point in which the error changes its trend. In other words, the error decreases until it has the three closest samples, then, the error begins to increase.

This happens because the method obtains relative distances from the samples to the sensor and when the method begins to use measures that are not close to the sensor the error increases. Obviously, the smaller the area is, where the sensor can be found, the lower the error in its location will be. More samples will give higher relative distances and therefore the error of location will be greater.

Our first conclusion, based on the previous graph, is that given a fixed number of samples, there will be a value of number of samples where the location error will be the optimal. Then, if the number of samples used to train the system is greater, the estimated position will be more accurate because there will be closer samples.

### 4.3. Error Measurement Based on the number of APs (Base Stations)

**Figure 4.**
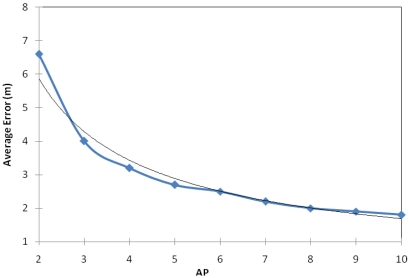
Average location estimation error as a function of the number of APs.

In order to test the influence of the number of APs in our proposal, we measured the error of the approach adding access point one by one in each location (in the same place of the 56 samples previously taken). In Figure 4 we can observe that the localization error tends to decrease exponentially (blue line with squares). Therefore, with higher number of APs we obtain lower error values in the sensor location estimation. This tendency is given because one of the methods used in our hybrid system is based on the triangulation method. This method uses the distance from the sensor to various access points based on RSS. Once the sensor obtains the value of at least three distances, between the sensor and the APs, the sensor estimates its position. Therefore, the more distances the sensor to different APs has, the higher the accuracy of the localization sensor will be, in other words, the error of location will be lower.

Bearing in mind this tendency, we have estimated which function follows the error when the number of APs of the indoor environment varies. Equation (10) gives our estimation:

(10) $$\text{y}=2,8383{\text{e}}^{-0,255\text{x}}$$

where *x* is the number of APs in the indoor environment. Equation (10) is shown in figure 4 with the black-thin line. We can see that it fits the error tend quite well.

It should be noted, that when there are more than five APs the improvement appreciation in terms localization error is minimal when a new AP is added to the indoor environment.

### 4.4. Comparative Measurement with others Existing Location Systems

In order to compare our proposal with others, we have evaluated five wireless sensor location systems:

- Inductive 1. This is an inductive location system which has enough a number of samples for an adequate training.
- Inductive 2. It is also an inductive location system, but in this case, the number of samples is very low.
- Deductive. This system uses the method based on the equation of spread that we have seen in subsection 4.3.
- The Hybrid method. This is our proposed method.
- Ekahau, which is the basis of many currently used location systems [38].

For the inductive methods we used a system described in our previous work [11]. As has been previously mentioned, inductive methods need a training phase. For the Inductive 1 and Ekahau methods, we collected 312 samples spread equitably throughout the floor of the building. The floor was split in a grid where sampling points are placed every 2 meters. Thirty observations were taken from each training point; 15 of them were taken one day and the other 15 were taken one week later. In contrast, for the Inductive 2 and hybrid methods we used a subset of 56 samples. For the hybrid method we estimated p(*o_i_*|*l*) using the histogram method shown in references [30,32].

**Figure 5.**
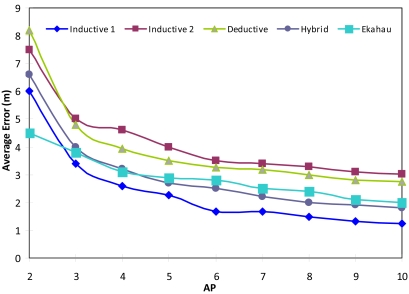
Comparative of average location error.

In the test phase, all these systems were tested for 40 locations (all these locations were different that the training ones, they were randomly placed and they were not inside the training grid). For each location we gathered a mean of 15 RSS consecutive values. This let us take into account the signal variability in the measurements. Each one of the test samples has been applied to the different location methods. Then, we estimate the error measuring the Euclidean distance between the output of the method and the real location of the sample. Figure 5 shows the results obtained for all the location systems as a function of the number of APs. Their graph follows an exponential tends approximately.

We note that the Inductive 2 method has a higher localization error than the others. This method had 56 training samples. They were few compared with the Inductive 1 method (312 samples). This difference gives considerably more accuracy in the Inductive 1 than the Inductive 2 method.

With regards to the deductive model, we note that it did not give good results because the floor where the measurements were taken had many walls, so there was very little accuracy when we estimated their loss.

The hybrid model proposed in this paper has a stable and optimal graph compared to the rest of systems (with few training measures low errors were obtained). As noted in Figure 5, for a certain number of AP (five APs) its average error remains the second best.

Finally, the Ekahau system together with the Inductive 2 system are the methods with the worst results.

In Table 2 we can see the average error and the standard deviation of the approached compared in our experiment. We see that the method with less error is the Inductive 1 (1.23 m), this is because the number of samples is adequate. The model with the worst behaviour is the Inductive 2 (3.02 m). The proposed hybrid method has an average error of 1.80 m, with the advantage that training is minimal. A statistical significance test has been calculated using a paired t-test (the hybrid approach is used as reference). A result labelled with a “^▲^”means statistical confidence of 99%. “^Δ^” means statistical confidence of 90%.

## 5. Hybrids Methods Comparison

This section compares our proposal with the hybrid methods found in the literature. In Table 3 we can see the performance analysis. First, we analyzed the analytical techniques used. All cases use multiple parameters, except in [28,29] and our proposal. The next feature compared was their working environment (indoor or outdoor environments). Our proposal and the one in reference [28] support both environments. The systems used in WSNs are [26,27] and our application; the others are used for other purposes. Our location system has a better accuracy (1.8 m) than other works, although the systems in [27, 28 and 29] have good features too.

The next analyzed feature is the number of stages to ascertain the final position. In this case, our system has two stages. The best solution is one stage because of simplicity. As we can see in Table 3, only the systems in [26] and [27] have one stage, but these systems need extra messages to estimate the location. But, on the other hand there are several works that demonstrate that sending messages wastes more energy than computing [39]. Finally we have analyzed the centralization and decentralization of the location systems and if they are recursively updated. Only our proposal and the one in reference [28] are recursively updated. In conclusion, we can see that the proposed location system has more positive features and it improves all analyzed hybrid location systems.

## 6. Conclusions

In this paper two different approaches had been discussed. The so-called deductive methods, which require a model of propagation, a topological information of the environment, and the exact position of the radio stations, and the so-called inductive methods, which require a previous training phase where the system learns the signal strength in each location. The main shortcoming of this approach is that in some scenarios, the training phase cannot be done or could be very expensive. On the other hand, the Euclidean models are optimum when there are multiple access points and few walls.

In this work we present a hybrid location system using a new stochastic approach which is based on a combination of deductive and inductive methods. This system has been developed for wireless sensor networks using IEEE 802.11b/g standard in order to use a deployed wireless access network that is also used for internet access and data transfer.

We have tested the errors based on the number of samples and based on the number of base stations. We have estimated an experimental equation based on the graph trends of the errors of the measures obtained. We have compared our proposal with several methods in order to check our approach.

Our system uses a small set of training samples (inductive information). Given the actual signal strength, we use the closest training samples as a starting point. Then, the deductive propagation model is used to obtain the shift from the training samples. A stochastic approach is used whereby the optimal location can be estimated as the point that maximizes the product of probabilities from each of the closest training samples

Our proposal combines the advantages of the deductive and inductive methods in order to provide accurate measurements in hard environments (few base stations and/or few trained points). The goal of this work has been to reduce the training phase without losing precision. Now, we are trying to find the proposed model by adding other methods in order to obtain more accurate results.

The proposed method is useful in cases where a good training phase is not practical (very few samples can be taken in advance), and the precise location of some access points is not known. These environments could be military, such as troop deployments inside buildings or discovery squads for hard environments, environments where the radio coverage is not known in advance (unknown deployments), or even environments where there the APs can be on or off at any time (dynamic environments). We are currently working on enhancing the precision of the proposed model. In future work we will evaluate the performance our proposal in hard environments.

## Figures and Tables

**Table 1.** Variables.

**Parameter**	**Description**	**Parameter**	**Description**
***l***	found location	***l****^j^*	location of base-station *j*
***o***	current observation	***d****_0_*	reference distance for signal strength measured
***b***	number of base station	***o****_0_^j^*	mean signal strength measured to *d_0_* distance from base-station *j*
***o****^j^*	signal strength from base-station *j* of *o; j*∈{1,…,*b*}	***d****^j^*	Euclidian distance between *l* and *l^j^*
***T***	set of training data	***n***	attenuation variation index
***t***	number of training samples	***L****_w_^j^*	attenuation caused by the obstacles from base-station *j*
***T****_i_*	training sample *i; i*∈{1,…,*t*}; *T_i_* = (*l_i_*, *o_i_*)	***w***	number of wall crossed
***l****_i_*	location of training sample *i*	***L****_0_*	wall average attenuation
***o****_i_*	observation of training sample *i*	***X****_σ_*	zero-mean normal distributed random variable with standard deviation σ.
***o****_i_^j^*	signal strength of training sample *i* from base-station *j*	***d****_i_^j^*	distance from *l_i_* to *l^j^*
***B****^j^*	base-station *j; j*∈{1,…,*b*}; *B^j^* = (*l^j^*, *o_0_^j^*)	***L****_wi_^j^*	wall attenuation obtained from *l_i_* to *l^j^*

**Table 2.** Average errors and standard deviation to the surveyed approaches.

	**Inductive 1**	**Inductive 2**	**Deductive**	**Hybrid**	**Ekahau**
**Error (m)**	1.23^▲^	3.02^▲^	2.75^▲^	1.80	2.04^Δ^
**Standard deviation**	0.62	2.12	1.73	0.74	1.61

**Table 3.** Hybrid location systems comparison.

	**Gu *et al.*** [24]	**McGuire *et al.*** [25]	**Catovic *et al.*** [26]	**Sahinoglu *et al.*** [27]	**Wang *et al.*** [28]	**Li *et al.*** [29]	**Our system**
***Analytical techniques***	*AOA/TOA*	*RSS/TDOA*	*TOA/RSS and TDOA/RSS*	*TDOA/RSS*	*RSS*	*RSS*	*RSS*
***Outdoor or indoor***	*Outdoor*	*Outdoor*	*Outdoor*	*Outdoor*	*Both*	*Indoor*	*Both*
***Is it used in WSNs?***	*No*	*No*	*Yes*	*Yes*	*No*	*No*	*Yes*
***Mean accuracy (m)***	*122*	*60*	*2-3.3*	*2.1*	*1.86*	*1.83*	*1.8*
***Stages***	*1*	*1*	*1*	*1*	*3*	*2*	*2*
***Extra messages***	*No*	*No*	*Yes*	*Yes*	*No*	*No*	*No*
***Centralized or distributed***	*Centralized*	*Centralized*	*Both*	*Distributed*	*Centralized*	*Centralized*	*Centralized*
***Recursively update***	*No*	*No*	*No*	*No*	*Yes*	*No*	*Yes*
